# Supplementary material for: Enabling work participation for people with musculoskeletal conditions: lessons from work changes imposed by COVID-19: a mixed-method study
Source: BMJ Open. 2022 Apr 7;12(4):e057919. doi: 10.1136/bmjopen-2021-057919 (PMC8990257; doi:10.1136/bmjopen-2021-057919)
Supplement: Author's manuscript [file bmjopen-2021-057919.draft_revisions.pdf]

BMJ Open is committed to open peer review. As part of this commitment we make the peer review history of every article we publish publicly available.

When an article is published we post the peer reviewers' comments and the authors' responses online. We also post the versions of the paper that were used during peer review. These are the versions that the peer review comments apply to.

The versions of the paper that follow are the versions that were submitted during the peer review process. They are not the versions of record or the final published versions. They should not be cited or distributed as the published version of this manuscript.

BMJ Open is an open access journal and the full, final, typeset and author-corrected version of record of the manuscript is available on our site with no access controls, subscription charges or pay-per-view fees (http://bmjopen.bmj.com).

If you have any questions on BMJ Open's open peer review process please email info.bmjopen@bmj.com

BMJ Open

Enabling work participation for people with musculoskeletal conditions. Lessons from work changes imposed by COVID-19: a qualitative study

Journal:	*BMJ Open*
Manuscript ID	bmjopen-2021-057919
Article Type:	Original research
Date Submitted by the Author:	30-Sep-2021
Complete List of Authors:	Morton, Lakrista; University of Aberdeen School of Medicine Medical Sciences and Nutrition, Epidemiology Group; University of Aberdeen School of Medicine Medical Sciences and Nutrition, Medical Research Council Versus Arthritis Centre for Musculoskeletal Health and Work<br>Stelfox, Kevin; University of Aberdeen School of Medicine Medical Sciences and Nutrition, Epidemiology Group; University of Aberdeen School of Medicine Medical Sciences and Nutrition, Medical Research Council Versus Arthritis Centre for Musculoskeletal Health and Work<br>Beasley, Marcus; University of Aberdeen School of Medicine Medical Sciences and Nutrition, Aberdeen Centre for Arthritis and Musculoskeletal Health (Epidemiology Group); University of Aberdeen School of Medicine Medical Sciences and Nutrition, Medical Research Council Versus Arthritis Centre for Musculoskeletal Health and Work<br>Jones, Gareth; University of Aberdeen School of Medicine Medical Sciences and Nutrition, Aberdeen Centre for Arthritis and Musculoskeletal Health (Epidemiology Group); University of Aberdeen School of Medicine Medical Sciences and Nutrition, Medical Research Council Versus Arthritis Centre for Musculoskeletal Health and Work<br>Macfarlane, Gary; University of Aberdeen School of Medicine Medical Sciences and Nutrition, Aberdeen Centre for Arthritis and Musculoskeletal Health (Epidemiology Group); University of Aberdeen School of Medicine Medical Sciences and Nutrition, Medical Research Council Versus Arthritis Centre for Musculoskeletal Health and Work<br>Walker-bone, Karen; University of Southampton, Medical Research Council Versus Arthritis Centre for Musculoskeletal Health and Work<br>Hollick, Rosemary; University of Aberdeen School of Medicine Medical Sciences and Nutrition, Aberdeen Centre for Arthritis and Musculoskeletal Health (Epidemiology Group); University of Aberdeen School of Medicine Medical Sciences and Nutrition, Medical Research Council Versus Arthritis Centre for Musculoskeletal Health and Work
Keywords:	QUALITATIVE RESEARCH, RHEUMATOLOGY, OCCUPATIONAL & INDUSTRIAL MEDICINE

I, the Submitting Author has the right to grant and does grant on behalf of all authors of the Work (as defined in the below author licence), an exclusive licence and/or a non-exclusive licence for contributions from authors who are: i) UK Crown employees; ii) where BMJ has agreed a CC-BY licence shall apply, and/or iii) in accordance with the terms applicable for US Federal Government officers or employees acting as part of their official duties; on a worldwide, perpetual, irrevocable, royalty-free basis to BMJ Publishing Group Ltd ("BMJ") its licensees and where the relevant Journal is co-owned by BMJ to the co-owners of the Journal, to publish the Work in this journal and any other BMJ products and to exploit all rights, as set out in our licence.

The Submitting Author accepts and understands that any supply made under these terms is made by BMJ to the Submitting Author unless you are acting as an employee on behalf of your employer or a postgraduate student of an affiliated institution which is paying any applicable article publishing charge ("APC") for Open Access articles. Where the Submitting Author wishes to make the Work available on an Open Access basis (and intends to pay the relevant APC), the terms of reuse of such Open Access shall be governed by a Creative Commons licence – details of these licences and which Creative Commons licence will apply to this Work are set out in our licence referred to above.

Other than as permitted in any relevant BMJ Author's Self Archiving Policies, I confirm this Work has not been accepted for publication elsewhere, is not being considered for publication elsewhere and does not duplicate material already published. I confirm all authors consent to publication of this Work and authorise the granting of this licence.

For peer review only

Running head: Lessons for work participation

Title: Enabling work participation for people with musculoskeletal conditions. Lessons from work changes imposed by COVID-19: a qualitative study

LaKrista Morton[1,2,3], Kevin Stelfox[1,2,3], Marcus Beasley[1,2,3], Gareth T. Jones[1,2,3], Gary J. Macfarlane[1,2,3], Karen Walker-Bone[4], Rosemary Hollick[1,2,3]

[1]Epidemiology Group, University of Aberdeen, UK; [2] Aberdeen Centre for Arthritis and Musculoskeletal Health, University of Aberdeen, UK; [3] Medical Research Council Versus Arthritis Centre for Musculoskeletal Health and Work, Aberdeen, UK; [4]Medical Research Council Versus Arthritis Centre for Musculoskeletal Health and Work, Southampton, UK

Sources of support: This work was supported by Versus Arthritis [Grant Number: 20748] and the British Society for Rheumatology. The funding for the original studies included were from Versus Arthritis (MAmMOTH) and the British Society for Rheumatology (BSRBR-AS and BSR-PsA). LaKrista Morton is funded through the Medical Research Council/Versus Arthritis Centre for Musculoskeletal Health and Work (Versus Arthritis Grant No. 20665).

L. Morton, Research Fellow, PhD; K. Stelfox, Research Fellow, PhD; M. Beasley, Study Co-ordinator, PhD; GT Jones, Reader, PhD; GJ Macfarlane, Clinical Chair in Epidemiology, Dean of Interdisciplinary Research and Research Impact, PhD; K Walker-Bone, Professor in Occupational Rheumatology, Honorary Consultant in Rheumatology, Director Versus Arthritis/MRC Centre for Musculoskeletal Health & Work, PhD; R Hollick, Senior Clinical Lecturer, Honorary Consultant in Rheumatology, PhD

Conflict of interest: The authors declare no conflicts of interest.

Corresponding author: Rosemary Hollick; Health Sciences Building (Rm 107), Foresterhill Campus, University of Aberdeen, AB25 2ZD. E: rhollick@abdn.ac.uk T: @AberdeenEpi

ORCIDiD: https://orcid.org/0000-0001-6558-7189

Key indexing terms: work, COVID-19, axial spondyloarthritis, psoriatic arthritis, pain

For peer review only - http://bmjopen.bmj.com/site/about/guidelines.xhtml

Abstract

Objectives: To understand what we can learn from the impact of the COVID-19 pandemic and lockdown about what enables work participation for people with inflammatory arthritis and chronic pain conditions.

Methods: Three established cohorts involving individuals with axial spondyloarthritis, psoriatic arthritis, and musculoskeletal pain completed a questionnaire between July and December 2020. A subset of respondents were selected for semi-structured interviews that explored the impact of lockdown and associated work restrictions.

Results: 491 people (52% female, median age 49 years) who were employed at the time of lockdown responded to the questionnaire. The qualitative analysis included 157 free-text comments on work from the questionnaire and data collected within 18 interviews.

Participants reported impacts on mental and physical health, and significant financial anxieties. The impact of work changes varied depending on individual and home circumstances. Some felt forced to ignore advice to shield and continue working. The flexibility offered by home working and changes in commuting enabled greater physical activity for some, while others missed the exercise normally undertaken as part of their commute. Others reported a constant need to be "present" online, which heightened anxiety and worsened musculoskeletal symptoms.

Conclusion: Lockdown showed that flexible working arrangements, which consider the positive and negative aspects of commuting, posture, movement and work environment matter for work participation, and can have wider benefits in terms of health and wellbeing for those with long-term MSK conditions. Incorporating these into new models of work will help make the workplace more equitable and inclusive for people with long-term MSK conditions.

For peer review only - http://bmjopen.bmj.com/site/about/guidelines.xhtml

Strengths and limitations of this study

- The study included people with both inflammatory and non-inflammatory MSK conditions in well-characterised clinical cohorts of 'real world' patients pre-defined by symptoms or diagnosis.
- Inflammatory arthritis and chronic pain are good exemplars of disability for work caused by a range of long-term conditions and findings may be applicable to a wider range of people trying to work with chronic health conditions.
- A number of respondents worked in professional/associate professional roles, and fewer of our participants worked in lower paid jobs.
- We conducted the study at a single time point during or after the lockdown, and therefore could not capture longitudinal data, including the longer-term impact of changes to working practices.

For peer review only - http://bmjopen.bmj.com/site/about/guidelines.xhtml

Introduction

Being in work that is safe, healthy, and which gives individuals some control of their work is good for physical and mental health (1). Employment brings meaning and purpose in life, better financial control and benefits for dependents (2). People with chronic musculoskeletal (MSK) conditions (inflammatory rheumatic diseases and chronic pain conditions) want to participate in work (3) but many struggle to be able to do so (4-7). The pain, fatigue, mobility impairment and functional loss associated with chronic MSK conditions are themselves challenges to work. However, people with MSK conditions also report that factors associated with the nature and type of their employment are also important: line manager and co-worker support; flexibility of working hours and practices; time off to attend healthcare appointments; support for travel to/from the workplace; availability of car-parking; autonomy over how and when to complete work tasks and availability of simple, practical workplace modifications (3).

In 2020, the global COVID-19 pandemic created unprecedented change to peoples' lives. In the UK, the first case was reported in January 2020 and such was the pace of transmission that a national lockdown was declared from 23rd March 2020. People were only permitted to leave home to attend work if they were designated "key workers" (e.g. health and social care, transport, communications, supermarkets). Working changed dramatically: most key workers found their jobs increasingly pressured; those who could work from home made that transition but many other workers were furloughed (paid 80% salary subsidised by government), had to change job or were made redundant.

Therefore, the working lives of most UK workers changed overnight. However, working lives were not the only things changed during lockdown: elective healthcare was temporarily suspended and then resumed only remotely; access to primary care was hampered; and some people with MSK conditions (particularly those with inflammatory rheumatic conditions treated with immunosuppressive therapies) were advised to "shield" (stay at home and not go to work, even if they were key workers). Leaving home to exercise was encouraged but restricted to one hour/day and most facilities (parks, swimming pools, gyms) were closed. Taking exercise became restricted to walking, running or cycling, all challenging for people with MSK conditions.

For peer review only - http://bmjopen.bmj.com/site/about/guidelines.xhtml

The impact of the pandemic does however provide opportunities to evaluate the impact of changed working practices on people with chronic MSK conditions who were working at the time of lockdown, with the objective of learning lessons (good and bad) about supporting work participation for people with these conditions. Therefore, we aimed to explore: (1) effects of lockdown on their work circumstances and (2) advantages and disadvantages of changes in work circumstances.

Methods

Study design

Qualitative study embedded within an observational questionnaire study.

Setting

Primary care and secondary care based rheumatology services.

Participants

Participants from three UK cohorts were included in this study (the CONTAIN Study). The methods of data collection for this study are published (8). Briefly, within the CONTAIN Study we included individuals meeting ASAS criteria for axial spondyloarthritis (axSpA; (9)) who were originally recruited from 83 clinical rheumatology services (sites) across the UK during 2012-2017 (BSRBR-AS register); patients meeting classification criteria for psoriatic arthritis currently being recruited from sites across the UK (CASPAR;(10) (BSR-PsA register); and people recruited from general practices in three Scottish health boards who had consulted with regional pain in primary care and other symptoms (sleep problems and somatic symptoms) 2016-2017 (MAmMOTH Study; (11)).

Participants completed a questionnaire for the CONTAIN Study, and those who consented to further contact were purposively selected for interview across the UK based on: gender, employment status; age; and nature of their MSK condition.

For peer review only - http://bmjopen.bmj.com/site/about/guidelines.xhtml

Data collection

Questionnaire: Data were collected by questionnaire starting June 2020, with reminders posted from September 2020. We collected sociodemographic characteristics including gender, age, and current (main) job and industry. Deprivation status was determined using postcodes with reference to either the population of Scotland (12), England (13), or Wales (14). Main job and industry were coded to 4-digit Office for National Statistics Occupational Classification (SOC) Hierarchy codes (15) (Table 1). Respondents indicating that they were in employment prior to lockdown were asked whether and how their job had changed since lockdown. They were asked to evaluate their financial security (how difficult they thought it would be to meet their financial commitments "this month" and "over the next 12 months" (0-10 Likert scale, 0='not at all worried' and 10='extremely worried').

Qualitative data: An open text question asked about individuals' perceptions and experiences (positive and negative) during the COVID-19 pandemic:
"The situation brought about the COVID-19 pandemic has brought challenges to many of us, but also perhaps some positive changes to day-to-day life. If you wish, please use this box to describe the main challenges that you have faced, and/or any positive changes that you've experienced."
Any free-text responses that referred to work/employment were analysed as part of the qualitative analysis.

Interviews were carried out by telephone and focussed on impacts of the pandemic and restrictions on employment, access to health care and on health. Work-related questions specifically aimed to gather insights about how people's work and ability to work had changed as a result of the pandemic. We also asked about changes to working practices, financial impacts, their employer's awareness of their condition, and any concerns about returning to work. The qualitative data collected as part of the CONTAIN study provided the opportunity "to generate a deep understanding of people's experiences, motivations, beliefs, goals, expectations, and needs" (16).

For peer review only - http://bmjopen.bmj.com/site/about/guidelines.xhtml

Data analysis

Of interest to the analysis presented in this manuscript are the CONTAIN study participants who were in paid employment immediately before the first UK lockdown. Using the questionnaire data, we investigated the sociodemographic characteristics of respondents based on their occupational status at the time: (a) those who remained working as usual (including key workers), (b) those who continued working but from home elsewhere, and (c) those who were furloughed, changed job or made redundant. The sociodemographic characteristics of these groups were explored using simple frequencies. Measures of financial concern were investigated across occupational groups.

Interviews were audio recorded and transcribed verbatim. Transcribed interviews and free-text responses from the questionnaire were uploaded into NVivo 12 software to facilitate organisation and analysis of the qualitative data. Qualitative data was analysed thematically by KS and LM, supported by RH. Deductive and inductively derived coding (17) was used to identify and categorise themes within higher-order themes informed from the topic guide which specifically aimed to facilitate an understanding of 1) individuals' lived experiences of working with a musculoskeletal condition during the pandemic and 2) to understand, from individuals' perspectives, the benefits and challenges posed to working routines and environments during lockdown. The analytical process involved familiarisation with data and initial coding; organisation of codes according to similarity of meaning; and development and review of themes and subthemes. Emerging analysis was discussed and developed with all authors. Data saturation was deemed to have been achieved through thematic and code-saturation (18).

Questionnaire data and semi-structured interview data were collected and analysed simultaneously, to provide an in-depth understanding of lived experiences of people with chronic MSK conditions working during lockdown and to learn, from changes made during the pandemic, about how to best support future work participation.

For peer review only - http://bmjopen.bmj.com/site/about/guidelines.xhtml

Ethics

Ethical approval for BSRBR-AS was from NRES Committee North East (County Durham and Tees Valley, Reference 11/NE/0374); BSR-PsA from West of Scotland REC 3 (Reference 18/WS/0126) and; MAmMOTH from NRES Committee South West (Cornwall and Plymouth, Reference 16/SW/0019). Informed consent was given by participants for publication of material.

Patient and public involvement

Support at work has been identified as a key priority by patients. Patients and patient organisations provided input into items asked in the questionnaire, design of the interview schedule and review of study documentation. Our patient partners also provided comment on the manuscript. We will continue to work with our patient partners to create a summary of findings and disseminate these via our patient organisation partners.

Results

In total, 1054 individuals completed a CONTAIN study questionnaire (596 from BSRBR-AS, 162 from BSR-PsA, and 296 from MAmMOTH), representing 29% of those contacted (27% BSRBR-AS; 26% BSR-PsA; 33% MAmMOTH). Of the 491 who were in paid employment before lockdown and had complete data, 51.7% were female (0.2% non-binary) with a median age of 49 years (range 21-75); 61.9% were from BSRBR-AS, 17.9% from BSRBR-PsA and 20.2% from MAmMOTH.

We included 157 responses from the free-text questionnaire item in the qualitative thematic analysis that referred to “work”. 57.3% of these respondents were from BSRBR-AS, 19.1% from BSR-PsA and 23.6% from MAmMOTH. 57.3% were female and had a median age of 47 years (range 27-78). 18 participants were interviewed who were in paid work immediately prior to the start of the first UK lockdown: 7 from BSRBR-AS, 7 from BSR-PsA and 4 from MAmMOTH. Eight were female, they were aged 28-64 years and lived in England (n=12), Scotland (n=5) and Wales (n=1). Of these 16 were Managers, directors (senior officials), professionals or in associate professional and technical occupations (Table 1).

For peer review only - http://bmjopen.bmj.com/site/about/guidelines.xhtml

Key themes identified within qualitative interviews and free-text questionnaire item responses are described within Table 2.

Changes to work status and circumstances

Table 3 summarises the changes to work status caused by lockdown of working questionnaire respondents. In total, 55% (n=268) continued to work as usual (most of whom, 76% (n=205) were key workers), 24% (n=120) changed to home working, and 21% (n=103) changed job/were furloughed/made redundant (Table 2). Older workers (aged >56 years) were those most likely to have been furloughed/changed job or made redundant (43.7%) and unsurprisingly no one working as process/plant/machine operatives, skilled tradesperson or in an elementary occupation was able to work from home.

The interviews provided insight into the changes to work status and the impact of these changes to health and financial circumstances. Some felt they had no choice but to continue working as usual, despite advice to shield.

"Me and my family being forced to work whilst most people were at home was stressful, my work not implementing safety procedures early or well enough was also a challenge. The only positive is that we haven't caught it." Q499 (Male; 40-55; SOC1)

A community care worker described how her company were unable to furlough her, despite advice to shield, which left her with no choice but to return to work.

Quite a few of the people I go to, like I say, they're elderly and they still have family members coming in and out to do their shopping. So, the people I've been going to haven't been fully isolating they haven't been able to so, just been fortunate I haven't picked it up. I16 (Female; ≤39; SOC6)

Others stopped taking their immunosuppressant medication (prescribed to control their inflammatory arthritis) so that they could continue working.

I stopped my medication for 2 months as I was very wary about taking it, I spoke to my rheumatologist first, I was also then allowed to continue to work, my employers were very

For peer review only - http://bmjopen.bmj.com/site/about/guidelines.xhtml

For peer review only

good and we had gone down to just 3 members of staff. Questionnaire 360 (Female; 40-55; SOC3)

For some, being self-employed enabled them to continue working in a way that suited them and their MSK condition.

I think because obviously I run my business, I've been able to be really flexible and work to suit myself, where if I'd been employed I don't know how that would have impacted me. I11 (Female; 40-55; SOC1)

Those who had lost their jobs explained that choices about alternative work would be influenced by their MSK condition, and might make them vulnerable to financial hardship.

Unfortunately I was made redundant due to COVID 19 and my work closed. I am looking to become self-employed but worried about money and if I can work as hard as I can. Q375 (Male; 40-55; Formerly SOC 4)

When asked about financial stability within the questionnaire, we found a wide distribution of responses, with generally more concerns expressed about finances in 12 months' time as compared with next month (Table 4).

Qualitative data collected from questionnaire free-text responses and the semi-structured interviews indicated that those who were unable to work from home generally reported more economic anxiety.

'they [employer] wouldn't furlough me, so they gave me… it was part sick pay, type thing…because I'm part time I don't earn enough to get full sick pay. I16 (Female; ≤39; SOC6)

Some respondents reported profound financial impact, exacerbating existing health worries and creating additional health problems.

The main challenge for me has been trying to survive financially. My job ended as soon as the lockdown was announced and I received only one final wage. I have not been eating

For peer review only - http://bmjopen.bmj.com/site/about/guidelines.xhtml

properly as I cannot afford to and this is making me very depressed. Q478 (Female, ≤39, SOC4).

I've had hospital appointments cancelled, new medication cancelled, financial loss of up to a third per month (approx £700 down per month) depression, severe weight gain. Q19 (Male; ≤39; SOC5)

Disadvantages and advantages of changes to work circumstances

Home working offered the opportunity for flexibility but the ability to take advantage of this depended upon individual and home circumstances. Those with partners who were working e.g. as key workers, and those with caring responsibilities, described difficulties juggling family and work responsibilities on top of their arthritis and recommendations to self-isolate.

The main challenge has been being stuck indoors or with limited access to outdoor space for months on end whilst at the same time looking after children (my wife is a key worker and has been at work), working a full-time job from home and dealing with my arthritis. I am certain that my arthritis is much more painful now in my hands, arms, shoulders, neck and back because of a poor working from home setup and also the lack of exercise I have been able to take. Q355 (Male; 40-55; SOC1)

Others who had previously used their commute to work as an opportunity for exercise were similarly affected by a more sedentary lifestyle, which had a negative impact on their MSK condition.

I've realised if you cycle to work, you don't feel so stiff at the end of it…if I did nothing, if I came down [stairs], started work, I would just stay stiff all day I think. I10 (Male; ≥56; SOC2*).*

Home workers reflected on work activities being more monotonous with an increase in more repetitive, computer-based tasks. For some, this led to an overall reduction in physical activity and more pain.

For peer review only - http://bmjopen.bmj.com/site/about/guidelines.xhtml

I'm not getting up and down as much as I would in the practice, I would be up and asking the GP a question, going to reception, go out to get a patient, having a chat with a nurse, so all of that movement has disappeared and I'm sitting all day…the lack of exercise probably has impacted more than I think. I4 (Female; 40-55; SOC2)

Home working also highlighted the role of trust between employees and employers. For some, not being physically present in the workplace environment created a sense of needing to be constantly 'present online' which negatively impacted on their MSK condition.

I'm finding myself sitting at my computer earlier and leaving it later at the end of the day…I've noticed that throughout a lot of the members of my team as well, they feel that if an email comes through you need to respond to it quickly or else people think you're being a slacker and that you're not at your desk and you're not working and stuff. I6 (Male; ≥56; SOC3)

Several participants also highlighted missing the social aspect of going to work, particularly for those who lived alone and were required to shield.

Living by myself in isolation has been a real test of my mental strength. Working from home and being without my work colleagues has also been challenging. Lack of social intersection has been the biggest challenge for me. Q9 (Female; ≤39; SOC2)

In contrast, some individuals e.g. those without home-schooling commitments, found it easier to take advantage of the flexibility offered by home working.

For some, less time spent commuting also provided a number of benefits such as reducing stress levels, freeing up time for other things including exercise, and improving arthritis symptoms, particularly back symptoms.

By not driving for 2 hours a day I got more of my work done and was able to walk for an hour a day which has helped my back and wellbeing. Q137 (Female; 40-55; SOC2)

A slower pace of life was helpful for many, facilitating regular rest and energy conservation and improving fatigue whilst still enabling them to do their job from home.

For peer review only - http://bmjopen.bmj.com/site/about/guidelines.xhtml

I've also been able to have hot water bottles and rest regularly when working at home whilst still being able to do my job. It slowed my life down in a positive way and made me realise I'd been rushing around trying to fit too much into each day before lockdown. Q289 (Female; ≤39; SOC1)

I found shielding for 12 weeks enabled me to have more energy every day. It also enabled me to do some home exercise without the feeling of being too exhausted from being at work. I felt I pushed myself a lot but in a great way. I feel now I am back at work, I am getting back into my old pattern and feel fatigued. Q6 (Female; ≤39; SOC 4).

Changing to home working suddenly meant that some did not have suitable equipment, so that they could not work effectively at their computer for prolonged periods, and developed increased pain and fatigue. However, people reported that they gradually developed different ways of working at home which facilitated regular movement throughout the day, and/or scheduled physical activity into their day, which was beneficial, but this required a conscious effort and took time.

I was kind of 'hot desking', whether that was in the kitchen or in the garden or if it was… it's good in some respects, either I was constantly moving around, so therefore it did my back a lot of good. I13 (Male; ≤39; SOC2)

One of the real nice things was I was able to go out for lunch with my daughter and we'd go on like a bike ride of something like that. We'd spend an hour out, which I didn't do during a normal working day which is something I think I'll take forward from it, is the stepping out of the building, there's massive sort of benefits for you. I13 (Male; ≤39; SOC2)

While some individuals missed social interactions at work, others felt that less contact with their colleagues and/or the work environment reduced their stress levels. In addition, some reflected that home working made their underlying health conditions less visible and hence, less stigmatising.

There are fewer people around= less stressed. I can park my car when I use it for work. I saw less of my irritating colleagues. Q467 (Non-binary; 40-55; SOC2)

For peer review only - http://bmjopen.bmj.com/site/about/guidelines.xhtml

I was suffering from mental health issues, and this made me needing to work from home and take time off..[I now] stand out less from my colleagues. Q39 (Male; 40-55; SOC2)

Discussion

In this study of people living and working with long-term MSK conditions, we have explored the effects of the lockdown caused by COVID-19 on changes in work status and circumstances, and the perceived impact of these changes on health and wellbeing. People who continued working reported significant anxiety about becoming infected at work and perceived unfairness compared to those who were furloughed, or could work at home. Many reported anxiety about finances and future chances of employment. In many cases, people found themselves working from home for the first time. Home working was not a panacea for all but offered some advantages and disadvantages in terms of impact on mental and physical health and physical activity. Most importantly, our findings point to some solutions to address the disability employment gap as we move beyond the pandemic to enable those working with MSK conditions to remain in work: suitable equipment; hybrid home working; flexibility; relationship with and support from managers; and minimising the need to travel in traditional commuter times.

However, there are some limitations to consider when interpreting these findings. A number of respondents worked in professional/associate professional roles, and fewer of our participants worked in lower paid jobs. We conducted the questionnaire and interviews at a single time point during or after the lockdown, and therefore could not capture longitudinal data, including the longer-term impact of changes to working practices. However, the study included people with both inflammatory and non-inflammatory MSK conditions in well-characterised cohorts of 'real world' patients (19) pre-defined by symptoms or diagnosis as opposed to convenience samples. Inflammatory arthritis and chronic pain are good exemplars of disability for work caused by the range of long-term conditions and therefore, these findings may be applicable to a wider range of people trying to work with chronic health conditions.

The COVID-19 pandemic has brought into sharp focus population inequalities in terms of the health, work, and finances of people (20). We found that, in those with MSK conditions, all

For peer review only - http://bmjopen.bmj.com/site/about/guidelines.xhtml

these impacts coalesced, causing a complex relationship between socio-economic status, vulnerability to COVID-19 and risk of work-related exposure. As in other studies, individuals aged >56 years made up the highest proportion of people who had changed job/were furloughed/made redundant during COVID-19 (21). This may have important implications for older people with MSK conditions wishing to return to work, as older workers in particular report more difficulties gaining employment after losing a job (21). Other general population studies have found that those working in more manual/lower paid work were less likely to be able to work from home (22). However, for individuals with MSK conditions on immunosuppressant medication who were advised to shield and work from home, we have shown those working in manual, lower paid jobs (often public-facing roles with a higher risk of work-based exposure) were less likely to be able do so. The financial impacts of this were significant if individuals could not be furloughed. People reported having no choice but to continue working and described the anxiety caused in consequence. Recent evidence indicates increased work-related exposure to COVID-19 in those unable to work from home and who are in closer proximity to other people or in direct contact with the public; often low-income jobs in service sectors, such as health or social care, transportation, cleaning and hospitality (23). A recent online survey of 2,003 disabled workers or workers who have a health condition or impairment and who were in work at the start of the pandemic in February 2021 by the Trades Union Congress (24), suggests that disability employment gap has widened as a result of COVID-19.

Many of the advantages and disadvantages of working from home highlighted by people with chronic MSK conditions in this study are similar to those reported by workers without health conditions during the pandemic (25). People learned to adapt and many were able to work effectively from home. People with MSK conditions valued flexibility to organise tasks and the freedom to make decisions about when they did their work from home. Similarly, 9 out of 10 workers in one national survey reported that they got at least as much, if not more, work done at home as in the office, with almost three quarters of employees saying that they wanted to adopt hybrid working arrangements in the future (25). For people with MSK conditions, we have already shown that commuting to work, and driving, can be a significant challenge for people with inflammatory arthritis (7, 26), and these findings suggest that, for many, reduced need for commuting was beneficial.

For peer review only - http://bmjopen.bmj.com/site/about/guidelines.xhtml

However, home working was not a panacea. Amongst those for whom exercise formed part of their daily commute, working from home worsened pain and stiffness. Moreover, some missed the sociability and benefits of collaboration offered by working in shared workspaces (25). Whilst many reported improvements in work-life balance, and more time for exercise, others struggled to balance working from home with domestic responsibilities and managing their MSK condition on top of this created additional burden. Interestingly, general population studies of workers have suggested people developed more musculoskeletal pain, and higher levels of fatigue and poor sleep early after lockdown, although things improved somewhat after a period of adjustment (25, 27).

Our study also highlighted the importance of the relationship between home-working employees and their managers. In a recent study of UK workers, participants who had more frequent contact with their line manager and had a work station risk assessment whilst home-working reported better mental health and less musculoskeletal pain (25). Line managers play a key role in supporting, motivating and engaging a remote or hybrid workforce, and employee wellbeing and line manager support is closely linked to productivity at work (25).

We already know that if people are out of the workplace for a long period that they are less likely to come back (28, 29). Loss of both routine and contact with the workplace has a detrimental impact on inactivity, isolation, well-being, as well as impaired self-image and career opportunities, physical fitness and confidence (30). Similarly, prolonged unemployment, for any reason, causes additional health problems. Those who lose their job suffer from worse mental health (31), poorer life expectancy (32), attend healthcare consultations more frequently with physical symptoms and report higher levels of pain (33). The significant numbers of people with long-term MSK conditions who were previously coping at work, and who have now found themselves out of the workplace for long periods because of the pandemic, may suffer similar consequences without additional support.

Conclusions

This study provides new insights into the impact of the COVID-19 pandemic on people living and working with MSK conditions within well-defined cohorts. It provides lessons to support those working with long-term MSK conditions, those with other long-term conditions and

For peer review only

the wider working population to work well and remain in work. Flexible working arrangements such as home-working can not only make the workplace more inclusive for people with disabilities and health conditions, but can also have wider benefits on their MSK health and wellbeing. These findings should encourage structural and organisational changes at the workplace to support people with long-term MSK conditions to work. There have however been concerns that individualised or complicated working patterns might not be sustainable within an organisation or might be perceived as unfair by co-workers (25, 34). However, flexibility and trust between employers and employees are the foundations of "good work" (35) and employers creating an open, flexible workplace experience better productivity and reduced staff turnover. The lockdown has provided a learning opportunity for both employees and employers to think creatively and shape new models of work that can accommodate everyone. The current study provides evidence of the value of some of these approaches when they are made available.

The pandemic has exposed pre-existing inequalities in socio-economic circumstances, health and work. Unfortunately, we have seen that people with long-term MSK conditions for whom flexibility was not possible, tended to be those from more socio-economically deprived backgrounds who have consequently been exposed to financial hardship or job loss or felt forced to ignore advice to shield and continue working, putting themselves at increased risk. Rheumatology services may see the consequences of this on work, health and finances for many years to come. However, some of the findings reported here may enable the rheumatology multi-disciplinary team to better support work participation for all patients with long-term MSK conditions and address the disability employment gap.

Data sharing statement

The data within the article which relate to the collection of BSR register data are owned by the BSR – access to these data are subject to application being made to the BSR: Registers (rheumatology.org.uk).

For peer review only

Contributorship statement

We are grateful to our patient partner Inga Wood for help with designing the interview schedule and for commenting on the manuscript and Lynne Laidlaw for help with designing questionnaire. The authors do not report any conflicts of interest. GJM conceived the idea for the study and all authors were involved in the detailed planning. LM, KS, and RH conducted the qualitative analysis with input from KWB. MB and GJ undertook the questionnaire analysis. LM and RH integrated questionnaire and quantitative findings, and KWB, KS and GJM contributed to interpretation of findings. RH and LM drafted the manuscript and all authors contributed important intellectual content via written comments.

For peer review only

For peer review only - http://bmjopen.bmj.com/site/about/guidelines.xhtml

References

1. Waddell GaB, A Kim. Is Work Good For Your Health and Well-Being? In: Pensions DfWa, editor.: The Stationary Office; 2006.
2. Black C. Working for a healthier tomorrow. Dame Carol Black's Review of the Health of Britain's Working Age Population. 2008.
3. Versus Arthritis. Working with Arthritis: Arthritis Research UK; 2016 [Available from: https://www.versusarthritis.org/media/2071/working-with-arthritis-policy-report.pdf.
4. Bridges S. Health Survey for England: Chapter 9 Chronic Pain. 2011. Contract No.: Report.
5. Price C, Hoggart B, Olukoga O, Williams A, Bottle A. National pain audit final report 2010–2012. London: Healthcare Quality Improvement Partnership, British Pain Society and Dr Foster Intelligence. 2012.
6. Macfarlane GJ, Shim J, Jones GT, Walker-Bone K, Pathan E, Dean LE. Identifying Persons with Axial Spondyloarthritis At Risk of Poor Work Outcome: Results from the British Society for Rheumatology Biologics Register. The Journal of rheumatology. 2019;46(2):145-52.
7. Hollick RJ, Stelfox K, Dean LE, Shim J, Walker-Bone K, Macfarlane GJ. Outcomes and treatment responses, including work productivity, among people with axial spondyloarthritis living in urban and rural areas: a mixed-methods study within a national register. Ann Rheum Dis. 2020;79(8):1055-62.
8. Macfarlane G, Hollick R, Morton L, Heddle M, Bachmair E, Anderson R, et al. The effect of COVID19 public health restrictions on the health of people with musculoskeletal conditions and symptoms: the CONTAIN study. . Rheumatology.
9. Rudwaleit M, van der Heijde D, Landewe R, Listing J, Akkoc N, Brandt J, et al. The development of Assessment of SpondyloArthritis international Society classification criteria for axial spondyloarthritis (part II): validation and final selection. Annals of the Rheumatic Diseases. 2009;68(6):777-83.
10. Taylor W, Gladman D, Helliwell P, Marchesoni A, Mease P, Mielants H. Classification criteria for psoriatic arthritis: development of new criteria from a large international study. Arthritis & Rheumatism: Official Journal of the American College of Rheumatology. 2006;54(8):2665-73.
11. Macfarlane GJ, Beasley M, Scott N, Chong H, McNamee P, McBeth J, et al. Maintaining musculoskeletal health using a behavioural therapy approach: a population-based randomised controlled trial (the MAmMOTH Study). Annals of the Rheumatic Diseases. 2021:annrheumdis-2020-219091.
12. Scottish index of multiple deprivation (SIMD) 2016.
13. English indices of deprivation (EID) 2015.
14. StatsWales. The Welsh Index of Multiple Deprivation 2014. .
15. Office for National S. SOC2010 volume 2: the structure and coding index.
16. Kelly A, Tymms K, Fallon K, Sumpton D, Tugwell P, Tunnicliffe D, et al. Qualitative Research in Rheumatology: An Overview of Methods and Contributions to Practice and Policy. J Rheumatol. 2021;48(1):6-15.
17. Braun V, Clarke V. Using thematic analysis in psychology. Qualitative Research in Psychology. 2006;3(2):77-101.
18. Braun V, Clarke V. To saturate or not to saturate? Questioning data saturation as a useful concept for thematic analysis and sample-size rationales. Qualitative Research in Sport, Exercise and Health. 2019;13(2):201-16.
19. Jones GT, Dean LE, Pathan E, Hollick RJ, Macfarlane GJ. Real-world evidence of TNF inhibition in axial spondyloarthritis: can we generalise the results from clinical trials? Annals of the Rheumatic Diseases. 2020;79:914-9.
20. Johnson P, Joyce R, Platt L. The IFS Deaton Review of Inequalities: a New Year's message. 2021. Contract No.: Report.
21. Bui TTM, Button P, Picciotti EG. Early Evidence on the Impact of Coronavirus Disease 2019 (COVID-19) and the Recession on Older Workers. Public Policy & Aging Report. 2020;30(4):154-9.

For peer review only - http://bmjopen.bmj.com/site/about/guidelines.xhtml

22. Adams-Prassl A, Boneva T, Golin M, Rauh C. Inequality in the impact of the coronovirus shock: new survey evidence for the UK. 2020. Contract No.: Report.
23. Industrial Injuries Advisory C. COVID-19 and occupation: IIAC position paper 48. 2021. Contract No.: Report.
24. Trades Union Congress (TUC). Disabled workers' experiences during the pandemic. A TUC Report 2021 [Available from: https://www.tuc.org.uk/sites/default/files/2021-06/Outline%20Report%20-%20Covid-19%20and%20Disabled%20Workers.pdf.
25. Parry J, Young Z, Bevan S, Veliziotis M, Baruch Y, Beigi M, et al. Working from Home under COVID-19 lockdown: Transitions and tensions, Work after Lockdown. 2021. Contract No.: Report.
26. Morton L, Macfarlane GJ, Jones G, Walker-Bone K, Hollick R. Driving difficulties in patients with axial spondyloarthritis: Results from the Scotland Registry for Ankylosing Spondylitis. Arthritis Care & Research. 2021.
27. Pieh C, Budimir S, Probst T. The effect of age, gender, income, work, and physical activity on mental health during coronavirus disease (COVID-19) lockdown in Austria. Journal of psychosomatic research. 2020;136:110186.
28. Black CaFD. Health at work - an independent review of sickness absence: The Stationery Office Limited; 2011 [Available from: https://assets.publishing.service.gov.uk/government/uploads/system/uploads/attachment_data/file/181060/health-at-work.pdf.
29. Waddell G, Burton AK. Occupational health guidelines for the management of low back pain at work: evidence review. Occupational Medicine. 2001;51(2):124-35.
30. Gustafsson K, Marklund S. Consequences of sickness presence and sickness absence on health and work ability: A Swedish prospective cohort study. International Journal of Occupational Medicine and Environmental Health. 2011;24(2):153-65.
31. Kposowa AJ. Unemployment and suicide: a cohort analysis of social factors predicting suicide in the US National Longitudinal Mortality Study. Psychological medicine. 2001;31(1):127.
32. Voss M, Nylen L, Floderus B, Diderichsen F, Terry PD. Unemployment and early cause-specific mortality: a study based on the Swedish twin registry. AmJPublic Health. 2004;94(12):2155-61.
33. Mason V. The prevalence of back pain in Great Britain.: Office of Population Censuses and surveys, HMSO; 1994.
34. Colella A. Coworker distributive fairness judgments of the workplace accommodation of employees with disabilities. Academy of management Review. 2001;26(1):100-16.
35. Taylor M, Marsh G, Nicol D, Broadbent P. Good work: the Taylor review of modern working practices. 2017. Contract No.: Report.

For peer review only

Table 1. Office for National Statistics Standard Occupational Classification (SOC) examples

SOC code for quotes	Major SOC category	Examples of occupations and 4-digit SOC codes
1	Managers, directors and senior officials	Chief executive (1115), health service manager (1181), head of public relations (1134)
2	Professional occupations	Social scientist (2114), civil engineer (2121), information technology professional (2139), primary school teacher (2315)
3	Associate professional and technical occupations	Laboratory technician (3111), paramedic (3213), artist (3411), careers advisor (3564)
4	Administrative and secretarial occupations	Post office clerk (4123), receptionist (4216), office manager (4161)
5	Skilled trades occupations	Landscape gardener (5113), electrician (5241), chef (5434), florist (5443)
6	Caring, leisure and other service occupations	Teaching assistant (6125), veterinary nurse (6131), hairdresser (6221)
7	Sales and customer service occupations	Retail assistant (7111), telephone salesperson (7113), window dressers (7125)
8	Process, plant and machine operatives	Textile process operative (8113), quarry worker (8123), scaffolder (8141)
9	Elementary occupations	Farm worker (9111), packer/bottler (9134), cleaner (9233)

For peer review only

Table 2. Key themes identified within qualitative interviews and free-text questionnaire item responses

Primary themes of inquiry	Sub-themes
**Changes to work status and circumstances**	• *Decisions about remaining at work*<br>• *Decisions about treatments*<br>• *Loss of earning*<br>• *Flexibility*
**Disadvantages and advantages of changes to work circumstances**	• *Managing multiple roles within the family*<br>• *Maintaining physical activity*<br>• *Working at home*<br>• *Workstation set up and ergonomics*<br>• *Relationship with employer*<br>• *Making adaptations*<br>• *Changes in pace of life*<br>• *Social interactions*<br>• *Stigma*

For peer review only

Table 3. Changes to individuals' work due to the pandemic, by sociodemographic factors (n=491)

		Continued working as usual, n (%)	[*keyworkers, n (%)*]	Working from home, n (%)	Furloughed/made redundant/changed job, n (%)
**Gender**	**Male**	126 (47.0)	*87 (42.4)*	54 (45.0)	56 (54.4)
	**Female**	141 (52.6)	*117 (57.1)*	66 (55.0)	47 (45.6)
	**Non-binary**	1 (0.4)	*1 (0.5)*	0	0
**Age**	**39 and under**	56 (20.9)	*45 (22.0)*	33 (27.5)	23 (22.3)
	**40 to 55**	133 (49.6)	*104 (50.7)*	60 (50.0)	35 (34.0)
	**56 and over**	79 (29.5)	*56 (27.3)*	27 (22.5)	45 (43.7)
**Job Type**	**Managers, directors, and senior officials**	31 (11.6)	*16 (7.8)*	20 (16.7)	18 (17.5)
	**Professional occupations**	98 (36.6)	*85 (41.5)*	55 (45.8)	16 (15.5)
	**Associate professional and technical**	32 (11.9)	*23 (11.2)*	17 (14.2)	17 (16.5)
	**Administrative and secretarial**	23 (8.6)	*18 (8.8)*	25 (20.8)	7 (6.8)
	**Skilled trades occupations**	19 (7.1)	*9 (4.4)*	0	14 (13.6)
	**Caring, leisure and other services**	23 (8.6)	*20 (9.8)*	1 (0.8)	7 (6.8)
	**Sales and customer service**	10 (3.7)	*9 (4.4)*	2 (1.6)	8 (7.8)
	**Process, plant and machine operatives**	13 (4.9)	*10 (4.9)*	0	9 (8.7)
	**Elementary Occupations**	18 (6.7)	*15 (7.3)*	0	7 (6.8)
	**Missing/NA**	1 (0.4)	*0*	0	0
**Deprivation**	**1 – Most deprived**	28 (10.4)	*24 (11.7)*	4 (3.3)	11 (10.7)
	**2**	37 (13.8)	*27 (13.2)*	14 (11.7)	15 (14.6)
	**3**	57 (21.3)	*44 (21.5)*	25 (20.8)	28 (27.2)
	**4**	80 (29.9)	*61 (29.8)*	32 (26.7)	23 (22.3)
	**5 – Least deprived**	66 (24.6)	*49 (23.9)*	45 (37.5)	26 (25.2)
	**Total n**	268	*205*	120	103

For peer review only - http://bmjopen.bmj.com/site/about/guidelines.xhtml

Table 4: Perceived difficulty and worry about meeting financial commitments in the coming month and over the next 12 months by job type (n=520)

	Median score [IQR]	
**Occupation (n)**	Perceived difficulty to meet financial commitments "this month" (0=not at all difficult; 10=extremely difficult)	Worry about meeting financial commitments over the next 12 months (0=not at all worried; 10=extremely worried)
Managers, directors and senior officials (72)	0 [0-1]	2 [0-4]
Professional occupations (173)	0 [0-1]	1 [0-3]
Associate professionals and technical occupations (71)	1 [1-2]	2 [0-6]
Administrative and secretarial occupations (56)	1 [0-1]	1 [0-3]
Skilled trades occupations (36)	0.5 [0-4.5]	2 [0-5.5]
Caring, leisure and other service occupations (38)	0 [0-3]	2 [0-5]
Sales and customer service occupations (24)	1 [0-2]	2 [0.5-5]
Process, plant and machine operatives (23)	0 [0-6]	1 [0-6]
Elementary occupations (27)	0 [0-2]	0 [0-5]

For peer review only - http://bmjopen.bmj.com/site/about/guidelines.xhtml

BMJ Open

Enabling work participation for people with musculoskeletal conditions. Lessons from work changes imposed by COVID-19: a mixed method study

Journal:	*BMJ Open*
Manuscript ID	bmjopen-2021-057919.R1
Article Type:	Original research
Date Submitted by the Author:	11-Feb-2022
Complete List of Authors:	Morton, Lakrista; University of Aberdeen School of Medicine Medical Sciences and Nutrition, Epidemiology Group; University of Aberdeen School of Medicine Medical Sciences and Nutrition, Medical Research Council Versus Arthritis Centre for Musculoskeletal Health and Work<br>Stelfox, Kevin; University of Aberdeen School of Medicine Medical Sciences and Nutrition, Epidemiology Group; University of Aberdeen School of Medicine Medical Sciences and Nutrition, Medical Research Council Versus Arthritis Centre for Musculoskeletal Health and Work<br>Beasley, Marcus; University of Aberdeen School of Medicine Medical Sciences and Nutrition, Aberdeen Centre for Arthritis and Musculoskeletal Health (Epidemiology Group); University of Aberdeen School of Medicine Medical Sciences and Nutrition, Medical Research Council Versus Arthritis Centre for Musculoskeletal Health and Work<br>Jones, Gareth; University of Aberdeen School of Medicine Medical Sciences and Nutrition, Aberdeen Centre for Arthritis and Musculoskeletal Health (Epidemiology Group); University of Aberdeen School of Medicine Medical Sciences and Nutrition, Medical Research Council Versus Arthritis Centre for Musculoskeletal Health and Work<br>Macfarlane, Gary; University of Aberdeen School of Medicine Medical Sciences and Nutrition, Aberdeen Centre for Arthritis and Musculoskeletal Health (Epidemiology Group); University of Aberdeen School of Medicine Medical Sciences and Nutrition, Medical Research Council Versus Arthritis Centre for Musculoskeletal Health and Work<br>Walker-bone, Karen; University of Southampton, Medical Research Council Versus Arthritis Centre for Musculoskeletal Health and Work<br>Hollick, Rosemary; University of Aberdeen School of Medicine Medical Sciences and Nutrition, Aberdeen Centre for Arthritis and Musculoskeletal Health (Epidemiology Group); University of Aberdeen School of Medicine Medical Sciences and Nutrition, Medical Research Council Versus Arthritis Centre for Musculoskeletal Health and Work
**Primary Subject Heading**:	Rheumatology
Secondary Subject Heading:	Qualitative research, Occupational and environmental medicine, Rheumatology
Keywords:	QUALITATIVE RESEARCH, RHEUMATOLOGY, OCCUPATIONAL & INDUSTRIAL MEDICINE, COVID-19

SCHOLARONE™
Manuscripts

I, the Submitting Author has the right to grant and does grant on behalf of all authors of the Work (as defined in the below author licence), an exclusive licence and/or a non-exclusive licence for contributions from authors who are: i) UK Crown employees; ii) where BMJ has agreed a CC-BY licence shall apply, and/or iii) in accordance with the terms applicable for US Federal Government officers or employees acting as part of their official duties; on a worldwide, perpetual, irrevocable, royalty-free basis to BMJ Publishing Group Ltd ("BMJ") its licensees and where the relevant Journal is co-owned by BMJ to the co-owners of the Journal, to publish the Work in this journal and any other BMJ products and to exploit all rights, as set out in our licence.

The Submitting Author accepts and understands that any supply made under these terms is made by BMJ to the Submitting Author unless you are acting as an employee on behalf of your employer or a postgraduate student of an affiliated institution which is paying any applicable article publishing charge ("APC") for Open Access articles. Where the Submitting Author wishes to make the Work available on an Open Access basis (and intends to pay the relevant APC), the terms of reuse of such Open Access shall be governed by a Creative Commons licence – details of these licences and which Creative Commons licence will apply to this Work are set out in our licence referred to above.

Other than as permitted in any relevant BMJ Author's Self Archiving Policies, I confirm this Work has not been accepted for publication elsewhere, is not being considered for publication elsewhere and does not duplicate material already published. I confirm all authors consent to publication of this Work and authorise the granting of this licence.

For peer review only

Running head: Lessons for work participation

Title: Enabling work participation for people with musculoskeletal conditions. Lessons from work changes imposed by COVID-19: a mixed method study

LaKrista Morton[1,2,3], Kevin Stelfox[1,2,3], Marcus Beasley[1,2,3], Gareth T. Jones[1,2,3], Gary J. Macfarlane[1,2,3], Karen Walker-Bone[4], Rosemary Hollick[1,2,3]

[1]Epidemiology Group, University of Aberdeen, UK; [2] Aberdeen Centre for Arthritis and Musculoskeletal Health, University of Aberdeen, UK; [3] Medical Research Council Versus Arthritis Centre for Musculoskeletal Health and Work, Aberdeen, UK; [4]Medical Research Council Versus Arthritis Centre for Musculoskeletal Health and Work, Southampton, UK

Sources of support: This work was supported by Versus Arthritis [Grant Number: 20748] and the British Society for Rheumatology. The funding for the original studies included were from Versus Arthritis (MAmMOTH) and the British Society for Rheumatology (BSRBR-AS and BSR-PsA). LaKrista Morton is funded through the Medical Research Council/Versus Arthritis Centre for Musculoskeletal Health and Work (Versus Arthritis Grant No. 20665).

L. Morton, Research Fellow, PhD; K. Stelfox, Research Fellow, PhD; M. Beasley, Study Co-ordinator, PhD; GT Jones, Reader, PhD; GJ Macfarlane, Clinical Chair in Epidemiology, Dean of Interdisciplinary Research and Research Impact, PhD; K Walker-Bone, Professor in Occupational Rheumatology, Honorary Consultant in Rheumatology, Director Versus Arthritis/MRC Centre for Musculoskeletal Health & Work, PhD; R Hollick, Senior Clinical Lecturer, Honorary Consultant in Rheumatology, PhD

Conflict of interest: The authors declare no conflicts of interest.

Corresponding author: Rosemary Hollick; Health Sciences Building (Rm 107), Foresterhill Campus, University of Aberdeen, AB25 2ZD. E: rhollick@abdn.ac.uk T: @AberdeenEpi ORCIDiD: https://orcid.org/0000-0001-6558-7189

Key indexing terms: work, COVID-19, axial spondyloarthritis, psoriatic arthritis, pain

For peer review only - http://bmjopen.bmj.com/site/about/guidelines.xhtml

Abstract

Objectives: To understand what we can learn from the impact of the COVID-19 pandemic and lockdown about what enables work participation for people with inflammatory arthritis and chronic pain conditions.

Design: Qualitative interviews embedded within an observational questionnaire study of individuals with musculoskeletal (MSK) conditions.

Setting: UK primary care (general practices), and secondary care-based rheumatology services.

Participants: Individuals with axial spondyloarthritis, psoriatic arthritis, and musculoskeletal pain from three established cohorts completed an online/paper-based questionnaire (July-December 2020). A subset of respondents were selected for semi-structured interviews.

Primary and secondary outcome measures: The survey quantified the effects of lockdown on work circumstances. Qualitative interviews explored the impacts of these changes and the advantages and disadvantages of changes in work circumstances.

Results: 491 people (52% female, median age 49 years) who were employed at the time of lockdown responded to the questionnaire. The qualitative analysis included 157 free-text comments on work from the questionnaire and data collected within 18 interviews.

Participants reported impacts on mental and physical health, and significant financial anxieties. The impact of work changes varied depending on individual and home circumstances. Some felt forced to ignore advice to shield and continue working. The flexibility offered by home working and changes in commuting enabled greater physical activity for some, while others missed the exercise normally undertaken as part of their commute. Others reported a constant need to be "present" online, which heightened anxiety and worsened musculoskeletal symptoms.

Conclusion: Lockdown showed that flexible working arrangements, which consider the positive and negative aspects of commuting, posture, movement, and work environment matter for work participation, and can have wider benefits in terms of health and wellbeing

For peer review only - http://bmjopen.bmj.com/site/about/guidelines.xhtml

for those with long-term MSK conditions. Incorporating these into new models of work will help make the workplace more equitable and inclusive for people with long-term MSK conditions.

For peer review only

For peer review only - http://bmjopen.bmj.com/site/about/guidelines.xhtml

Strengths and limitations of this study

- The study included people with both inflammatory and non-inflammatory MSK conditions in well-characterised clinical cohorts of ‘real world’ patients pre-defined by symptoms or diagnosis.
- Inflammatory arthritis and chronic pain are good exemplars of disability for work caused by a range of long-term conditions and findings may be applicable to a wider range of people trying to work with chronic health conditions.
- A number of respondents worked in professional/associate professional roles, and fewer of our participants worked in lower paid jobs.
- We collected data from participants at a single time point , and therefore could not capture longitudinal data, including the longer-term impact of changes to working practices.

For peer review only

For peer review only - http://bmjopen.bmj.com/site/about/guidelines.xhtml

Introduction

Being in work that is safe, healthy, and which gives individuals some control of their work is good for physical, social and psychological health (1). Employment can contribute to an individual's personal identity, facilitates social relationships, and can bring meaning and purpose in life (1,2). It also brings better financial control and benefits for dependents (2). People with chronic musculoskeletal (MSK) conditions (inflammatory rheumatic diseases and chronic pain conditions) want to participate in work (3) but many struggle to be able to do so (4-7). The pain, fatigue, mobility impairment and functional loss associated with chronic MSK conditions are themselves challenges to work. However, people with MSK conditions also report that factors associated with the nature and type of their employment are also important: line manager and co-worker support; flexibility of working hours and practices; time off to attend healthcare appointments; support for travel to/from the workplace; availability of car-parking; autonomy over how and when to complete work tasks and availability of simple, practical workplace modifications (3).

In 2020, the global COVID-19 pandemic created unprecedented change to peoples' lives. In the UK, the first case was reported in January 2020, and such was the pace of transmission that a national lockdown was declared from 23rd March 2020. People were only permitted to leave home to attend work if they were designated "key workers" (e.g., health and social care, transport, communications, supermarkets). Working changed dramatically: most key workers found their jobs increasingly pressured; those who could work from home made that transition but many other workers were furloughed (paid 80% salary subsidised by government), had to change job or were made redundant.

Therefore, the working lives of most UK workers changed overnight. However, working lives were not the only things changed during lockdown: elective healthcare was temporarily suspended and then resumed only remotely; access to primary care was hampered; and some people with MSK conditions (particularly those with inflammatory rheumatic conditions treated with immunosuppressive therapies) were advised to "shield" (stay at home and not go to work, even if they were key workers). Leaving home to exercise was encouraged but restricted to one hour/day and most facilities (parks, swimming pools,

For peer review only - http://bmjopen.bmj.com/site/about/guidelines.xhtml

gyms) were closed. Taking exercise became restricted to walking, running or cycling, all challenging for people with MSK conditions.

The impact of the pandemic does however provide opportunities to evaluate the impact of changed working practices on people with chronic MSK conditions who were working at the time of lockdown, with the objective of learning lessons (good and bad) about supporting work participation for people with these conditions. Within three well-defined cohorts of individuals with inflammatory and non-inflammatory conditions who were in paid employment immediately before the first UK lockdown, we aimed to, firstly, quantify effects of lockdown on their work circumstances (those who remained in work as usual; those who continued working but from home/elsewhere; and those who were furloughed, changed job or made redundant) and describe the socio-demographic and clinical characteristics of people within each group. Secondly, we aimed to explore individual's experiences of changes to work circumstances and the advantages and disadvantages of these changes.

Methods

Study design

Qualitative study embedded within an observational questionnaire study of individuals with MSK conditions, which has been reported in line with the Standards for Reporting Qualitative Research (Supplementary File).

Setting

UK primary care (general practices), and secondary care-based rheumatology services.

Participants

Participants from three UK cohorts were included in this study (the CONTAIN Study). The methods of data collection for this study are published (8). Briefly, the cohorts comprising the CONTAIN Study included individuals meeting ASAS criteria for axial spondyloarthritis (axSpA; (9)) who were originally recruited from 83 clinical rheumatology services (sites) across the UK

For peer review only - http://bmjopen.bmj.com/site/about/guidelines.xhtml

during 2012-2017 (BSRBR-AS register); patients meeting classification criteria for psoriatic arthritis currently being recruited from sites across the UK (CASPAR;(10) (BSR-PsA register); and people recruited from general practices in three Scottish health boards who had consulted with regional pain in primary care and other symptoms (sleep problems and somatic symptoms) 2016-2017 (MAmMOTH Study; (11)).

Participants from these three cohorts completed a questionnaire as an additional follow-up for the CONTAIN Study.

Data collection

Questionnaire: Data were collected by questionnaire (online or by paper if requested) starting June 2020, with reminders posted from September 2020. The questionnaire comprised existing validated instruments and questions specific to individuals' experiences during the COVID-19 pandemic (8). Relevant aspects for the current analysis included sociodemographic characteristics including gender, age, and current (main) job and industry. Deprivation status was determined using postcodes with reference to either the population of Scotland (12), England (13), or Wales (14). Main job and industry were coded to 4-digit Office for National Statistics Occupational Classification (SOC) Hierarchy codes (15) (Table 1). Respondents indicating that they were in employment prior to lockdown were asked whether and how their job had changed since lockdown. They were asked to evaluate their financial security (how difficult they thought it would be to meet their financial commitments "this month" and "over the next 12 months" (0-10 Likert scale, 0='not at all worried' and 10='extremely worried'). Written consent was obtained at the time of survey completion.

An open text question asked about individuals' perceptions and experiences (positive and negative) during the COVID-19 pandemic:

"The situation brought about the COVID-19 pandemic has brought challenges to many of us, but also perhaps some positive changes to day-to-day life. If you wish, please use this box to describe the main challenges that you have faced, and/or any positive changes that you've experienced."

For peer review only - http://bmjopen.bmj.com/site/about/guidelines.xhtml

Qualitative data: We conducted semi-structured qualitative interviews with a sub-set of those who had completed the questionnaire and consented to further contact. Participants were purposively selected for interview across the UK based on information provided in the questionnaire: gender, employment status ((a) those who remained in work as usual, (b) those who continued working but from home elsewhere, and (c) those who were furloughed, changed job or made redundant); age; and nature of their MSK condition (inflammatory (axSpA, PsA) and non-inflammatory).

Interviews were carried out by telephone and focussed on impacts of the pandemic and restrictions on employment, access to health care and on health. For the purposes of the current analysis, we analysed interview transcripts from those who were currently working. Work-related questions specifically aimed to gather insights about how people’s work and ability to work had changed because of the pandemic. We also asked about changes to working practices, financial impacts, their employer’s awareness of their condition, and any concerns about returning to work.

Interviews were conducted by KS, a research fellow with significant experience in conducting qualitative interviews with people with experience of MSK conditions and chronic pain. At the time of scheduling the interview with each potential participant, KS discussed the reasons for doing the research and answered any questions. Consent was obtained prior to interview using a written consent guide and this was audio recorded. Interviews were audio recorded and transcribed verbatim.

In addition, any responses from the open-ended question in the questionnaire that referred to work/employment were analysed thematically as part of the qualitative analysis.

The qualitative data collected as part of the CONTAIN study provided the opportunity “to generate a deep understanding of people’s experiences, motivations, beliefs, goals, expectations, and needs” (16).

For peer review only - http://bmjopen.bmj.com/site/about/guidelines.xhtml

Data analysis

Of interest to the analysis presented in this manuscript are the CONTAIN study participants who were in paid employment immediately before the first UK lockdown. Using the questionnaire data, we investigated the sociodemographic characteristics of respondents based on their occupational status at the time: (a) those who remained working as usual (including key workers), (b) those who continued working but from home elsewhere, and (c) those who were furloughed, changed job, or made redundant. The sociodemographic characteristics of these groups were explored using simple frequencies. Measures of financial concern were investigated across occupational groups.

Interviews were audio recorded and transcribed verbatim. Transcribed interviews and free-text responses from the questionnaire were uploaded into NVivo 12 software to facilitate organisation and analysis of the qualitative data. Qualitative data was analysed thematically by KS and LM, supported by RH. Deductive and inductively derived coding (17) was used to identify and categorise themes within higher-order themes informed by the topic guide which specifically aimed to facilitate an understanding of individuals' lived experiences of working with a musculoskeletal condition during the pandemic and the benefits and challenges posed to working routines and environments during lockdown. The analytical process involved familiarisation with data and initial coding; organisation of codes according to similarity of meaning; and development and review of themes and subthemes. Emerging analysis was discussed and developed with all authors. Data saturation was deemed to have been achieved through thematic and code-saturation (18), which was discussed and determined between KS and LM.

Questionnaire data and semi-structured interview data were collected and analysed concurrently, to provide an in-depth understanding of lived experiences of people with chronic MSK conditions working during lockdown and to learn, from changes made during the pandemic, about how to best support future work participation.

For peer review only - http://bmjopen.bmj.com/site/about/guidelines.xhtml

Ethics

Ethical approval for BSRBR-AS was from NRES Committee North East (County Durham and Tees Valley, Reference 11/NE/0374); BSR-PsA from West of Scotland REC 3 (Reference 18/WS/0126) and; MAmMOTH from NRES Committee South West (Cornwall and Plymouth, Reference 16/SW/0019). Informed consent was given by participants for publication of material.

Patient and public involvement

Support at work has been identified as a key priority by patients. Patients and patient organisations provided input into items asked in the questionnaire, design of the interview schedule and review of study documentation. Our patient partners also provided comment on the manuscript. We will continue to work with our patient partners to create a summary of findings and disseminate these via our patient organisation partners.

Results

In total, 1054 individuals completed a CONTAIN study questionnaire (596 from BSRBR-AS, 162 from BSR-PsA, and 296 from MAmMOTH), representing 29% of those contacted (27% BSRBR-AS; 26% BSR-PsA; 33% MAmMOTH). Of the 491 who were in paid employment before lockdown and had complete data, 51.7% were female (0.2% non-binary) with a median age of 49 years (range 21-75); 61.9% were from BSRBR-AS, 17.9% from BSRBR-PsA and 20.2% from MAmMOTH.

We included 157 responses from the free-text questionnaire item in the qualitative thematic analysis that referred to “work”. 57.3% of these respondents were from BSRBR-AS, 19.1% from BSR-PsA and 23.6% from MAmMOTH. 57.3% were female and had a median age of 47 years (range 27-78). Of 782 questionnaire respondents who provided consent to be contacted about an interview, we interviewed 23 (18 of whom were in paid work immediately prior to the start of the first UK lockdown and therefore included in this qualitative analysis) and reached data saturation at this stage. Of the 18 interviewees who were in paid work immediately prior to the start of the first UK lockdown: 7 were from BSRBR-AS, 7 from BSR-PsA and 4 from MAmMOTH. Eight were female, they were aged 28-

For peer review only - http://bmjopen.bmj.com/site/about/guidelines.xhtml

For peer review only

64 years and lived in England (n=12), Scotland (n=5) and Wales (n=1). Of these 16 were Managers, directors (senior officials), professionals or in associate professional and technical occupations (Table 1).

Table 1. Office for National Statistics Standard Occupational Classification (SOC) examples

**SOC code for quotes**	**Major SOC category**	**Examples of occupations and 4-digit SOC codes**
1	Managers, directors and senior officials	Chief executive (1115), health service manager (1181), head of public relations (1134)
2	Professional occupations	Social scientist (2114), civil engineer (2121), information technology professional (2139), primary school teacher (2315)
3	Associate professional and technical occupations	Laboratory technician (3111), paramedic (3213), artist (3411), careers advisor (3564)
4	Administrative and secretarial occupations	Post office clerk (4123), receptionist (4216), office manager (4161)
5	Skilled trades occupations	Landscape gardener (5113), electrician (5241), chef (5434), florist (5443)
6	Caring, leisure and other service occupations	Teaching assistant (6125), veterinary nurse (6131), hairdresser (6221)
7	Sales and customer service occupations	Retail assistant (7111), telephone salesperson (7113), window dressers (7125)
8	Process, plant and machine operatives	Textile process operative (8113), quarry worker (8123), scaffolder (8141)
9	Elementary occupations	Farm worker (9111), packer/bottler (9134), cleaner (9233)

Key themes identified within qualitative interviews and free-text questionnaire item responses are described within Table 2.

For peer review only - http://bmjopen.bmj.com/site/about/guidelines.xhtml

Table 2. Key themes identified within qualitative interviews and free-text questionnaire item responses

Primary areas of inquiry	Themes
**Changes to work status and circumstances**	• *Decisions about remaining at work*<br>• *Decisions about treatments*<br>• *Loss of earning*<br>• *Flexibility*
**Disadvantages and advantages of changes to work circumstances**	• *Managing multiple roles within the family*<br>• *Maintaining physical activity*<br>• *Working at home*<br>• *Workstation set up and ergonomics*<br>• *Relationship with employer*<br>• *Making adaptations*<br>• *Changes in pace of life*<br>• *Social interactions*<br>• *Stigma*

Changes to work status and circumstances

Table 3 summarises the changes to work status caused by lockdown of working questionnaire respondents. In total, 55% (n=268) continued to work as usual (most of whom, 76% (n=205) were key workers), 24% (n=120) changed to home working, and 21% (n=103) changed job/were furloughed/made redundant (Table 3). Older workers (aged >56 years) were those most likely to have been furloughed/changed job or made redundant (43.7%) and unsurprisingly no one working as process/plant/machine operatives, skilled tradesperson or in an elementary occupation was able to work from home.

For peer review only

Table 3. Changes to individuals' work due to the pandemic, by sociodemographic factors (n=491)

		**Continued working as usual, n (%)**	***[keyworkers, n (%)]***	**Working from home, n (%)**	**Furloughed/made redundant/changed job, n (%)**
**Gender**	**Male**	126 (47.0)	*87 (42.4)*	54 (45.0)	56 (54.4)
	**Female**	141 (52.6)	*117 (57.1)*	66 (55.0)	47 (45.6)
	**Non-binary**	1 (0.4)	*1 (0.5)*	0	0
**Age**	**39 and under**	56 (20.9)	*45 (22.0)*	33 (27.5)	23 (22.3)
	**40 to 55**	133 (49.6)	*104 (50.7)*	60 (50.0)	35 (34.0)
	**56 and over**	79 (29.5)	*56 (27.3)*	27 (22.5)	45 (43.7)
**Job Type**	**Managers, directors, and senior officials**	31 (11.6)	*16 (7.8)*	20 (16.7)	18 (17.5)
	**Professional occupations**	98 (36.6)	*85 (41.5)*	55 (45.8)	16 (15.5)
	**Associate professional and technical**	32 (11.9)	*23 (11.2)*	17 (14.2)	17 (16.5)
	**Administrative and secretarial**	23 (8.6)	*18 (8.8)*	25 (20.8)	7 (6.8)
	**Skilled trades occupations**	19 (7.1)	*9 (4.4)*	0	14 (13.6)
	**Caring, leisure and other services**	23 (8.6)	*20 (9.8)*	1 (0.8)	7 (6.8)
	**Sales and customer service**	10 (3.7)	*9 (4.4)*	2 (1.6)	8 (7.8)
	**Process, plant and machine operatives**	13 (4.9)	*10 (4.9)*	0	9 (8.7)
	**Elementary Occupations**	18 (6.7)	*15 (7.3)*	0	7 (6.8)
	**Missing/NA**	1 (0.4)	*0*	0	0
**Deprivation**	**1 – Most deprived**	28 (10.4)	*24 (11.7)*	4 (3.3)	11 (10.7)
	**2**	37 (13.8)	*27 (13.2)*	14 (11.7)	15 (14.6)
	**3**	57 (21.3)	*44 (21.5)*	25 (20.8)	28 (27.2)
	**4**	80 (29.9)	*61 (29.8)*	32 (26.7)	23 (22.3)
	**5 – Least deprived**	66 (24.6)	*49 (23.9)*	45 (37.5)	26 (25.2)
	**Total n**	268	*205*	120	103

For peer review only

For peer review only - http://bmjopen.bmj.com/site/about/guidelines.xhtml

The interviews provided insight into the changes to work status and the impact of these changes to health and financial circumstances. Some felt they had no choice but to continue working as usual, despite advice to shield.

"Me and my family being forced to work whilst most people were at home was stressful, my work not implementing safety procedures early or well enough was also a challenge. The only positive is that we haven't caught it." Q499 (Male; 40-55; SOC1)

A community care worker described how her company were unable to furlough her, despite advice to shield, which left her with no choice but to return to work.

Quite a few of the people I go to, like I say, they're elderly and they still have family members coming in and out to do their shopping. So, the people I've been going to haven't been fully isolating they haven't been able to so, just been fortunate I haven't picked it up. I16 (Female; ≤39; SOC6)

Others stopped taking their immunosuppressant medication (prescribed to control their inflammatory arthritis) so that they could continue working.

I stopped my medication for 2 months as I was very wary about taking it, I spoke to my rheumatologist first, I was also then allowed to continue to work, my employers were very good and we had gone down to just 3 members of staff. Questionnaire 360 (Female; 40-55; SOC3)

For some, being self-employed enabled them to continue working in a way that suited them and their MSK condition.

I think because obviously I run my business, I've been able to be really flexible and work to suit myself, where if I'd been employed I don't know how that would have impacted me. I11 (Female; 40-55; SOC1)

Those who had lost their jobs explained that choices about alternative work would be influenced by their MSK condition, and might make them vulnerable to financial hardship.

For peer review only - http://bmjopen.bmj.com/site/about/guidelines.xhtml

Unfortunately I was made redundant due to COVID 19 and my work closed. I am looking to become self-employed but worried about money and if I can work as hard as I can. Q375 (Male; 40-55; Formerly SOC 4)

When asked about financial stability within the questionnaire, we found a wide distribution of responses, with generally more concerns expressed about finances in 12 months' time as compared with next month (Table 4).

For peer review only

For peer review only - http://bmjopen.bmj.com/site/about/guidelines.xhtml

Table 4: Perceived difficulty and worry about meeting financial commitments in the coming month and over the next 12 months by job type (n=520)

	Median score [IQR]	
**Occupation (n)**	Perceived difficulty to meet financial commitments "this month" (0=not at all difficult; 10=extremely difficult)	Worry about meeting financial commitments over the next 12 months (0=not at all worried; 10=extremely worried)
Managers, directors and senior officials (72)	0 [0-1]	2 [0-4]
Professional occupations (173)	0 [0-1]	1 [0-3]
Associate professionals and technical occupations (71)	1 [1-2]	2 [0-6]
Administrative and secretarial occupations (56)	1 [0-1]	1 [0-3]
Skilled trades occupations (36)	0.5 [0-4.5]	2 [0-5.5]
Caring, leisure and other service occupations (38)	0 [0-3]	2 [0-5]
Sales and customer service occupations (24)	1 [0-2]	2 [0.5-5]
Process, plant and machine operatives (23)	0 [0-6]	1 [0-6]
Elementary occupations (27)	0 [0-2]	0 [0-5]

For peer review only

For peer review only - http://bmjopen.bmj.com/site/about/guidelines.xhtml

Qualitative data collected from questionnaire free-text responses and the semi-structured interviews indicated that those who were unable to work from home generally reported more economic anxiety.

'they [employer] wouldn't furlough me, so they gave me… it was part sick pay, type thing…because I'm part time I don't earn enough to get full sick pay. I16 (Female; ≤39; SOC6)

Some respondents reported profound financial impact, exacerbating existing health worries and creating additional health problems.

The main challenge for me has been trying to survive financially. My job ended as soon as the lockdown was announced and I received only one final wage. I have not been eating properly as I cannot afford to and this is making me very depressed. Q478 (Female, ≤39, SOC4).

I've had hospital appointments cancelled, new medication cancelled, financial loss of up to a third per month (approx £700 down per month) depression, severe weight gain. Q19 (Male; ≤39; SOC5)

Disadvantages and advantages of changes to work circumstances

Home working offered the opportunity for flexibility but the ability to take advantage of this depended upon individual and home circumstances. Those with partners who were working e.g. as key workers, and those with caring responsibilities, described difficulties juggling family and work responsibilities on top of their arthritis and recommendations to self-isolate.

The main challenge has been being stuck indoors or with limited access to outdoor space for months on end whilst at the same time looking after children (my wife is a key worker and has been at work), working a full-time job from home and dealing with my arthritis. I am certain that my arthritis is much more painful now in my hands, arms, shoulders, neck and

For peer review only - http://bmjopen.bmj.com/site/about/guidelines.xhtml

back because of a poor working from home setup and also the lack of exercise I have been able to take. Q355 (Male; 40-55; SOC1)

Others who had previously used their commute to work as an opportunity for exercise were similarly affected by a more sedentary lifestyle, which had a negative impact on their MSK condition.

I've realised if you cycle to work, you don't feel so stiff at the end of it…if I did nothing, if I came down [stairs], started work, I would just stay stiff all day I think. I10 (Male; ≥56; SOC2*).*

Home workers reflected on work activities being more monotonous with an increase in more repetitive, computer-based tasks. For some, this led to an overall reduction in physical activity and more pain.

I'm not getting up and down as much as I would in the practice, I would be up and asking the GP a question, going to reception, go out to get a patient, having a chat with a nurse, so all of that movement has disappeared and I'm sitting all day…the lack of exercise probably has impacted more than I think. I4 (Female; 40-55; SOC2)

Home working also highlighted the role of trust between employees and employers. For some, not being physically present in the workplace environment created a sense of needing to be constantly 'present online' which negatively impacted on their MSK condition.

I'm finding myself sitting at my computer earlier and leaving it later at the end of the day…I've noticed that throughout a lot of the members of my team as well, they feel that if an email comes through you need to respond to it quickly or else people think you're being a slacker and that you're not at your desk and you're not working and stuff. I6 (Male; ≥56; SOC3)

Several participants also highlighted missing the social aspect of going to work, particularly for those who lived alone and were required to shield.

For peer review only - http://bmjopen.bmj.com/site/about/guidelines.xhtml

Living by myself in isolation has been a real test of my mental strength. Working from home and being without my work colleagues has also been challenging. Lack of social intersection has been the biggest challenge for me. Q9 (Female; ≤39; SOC2)

In contrast, some individuals e.g., those without home-schooling commitments, found it easier to take advantage of the flexibility offered by home working.

For some, less time spent commuting also provided several benefits such as reducing stress levels, freeing up time for other things including exercise, and improving arthritis symptoms, particularly back symptoms.

By not driving for 2 hours a day I got more of my work done and was able to walk for an hour a day which has helped my back and wellbeing. Q137 (Female; 40-55; SOC2)

A slower pace of life was helpful for many, facilitating regular rest and energy conservation and improving fatigue whilst still enabling them to do their job from home.

I've also been able to have hot water bottles and rest regularly when working at home whilst still being able to do my job. It slowed my life down in a positive way and made me realise I'd been rushing around trying to fit too much into each day before lockdown. Q289 (Female; ≤39; SOC1)

I found shielding for 12 weeks enabled me to have more energy every day. It also enabled me to do some home exercise without the feeling of being too exhausted from being at work. I felt I pushed myself a lot but in a great way. I feel now I am back at work, I am getting back into my old pattern and feel fatigued. Q6 (Female; ≤39; SOC 4).

Changing to home working suddenly meant that some did not have suitable equipment, so that they could not work effectively at their computer for prolonged periods, and developed increased pain and fatigue. However, people reported that they gradually developed different ways of working at home which facilitated regular movement throughout the day, and/or scheduled physical activity into their day, which was beneficial, but this required a conscious effort and took time.

For peer review only

I was kind of 'hot desking', whether that was in the kitchen or in the garden or if it was… it's good in some respects, either I was constantly moving around, so therefore it did my back a lot of good. I13 (Male; ≤39; SOC2)

One of the real nice things was I was able to go out for lunch with my daughter and we'd go on like a bike ride of something like that. We'd spend an hour out, which I didn't do during a normal working day which is something I think I'll take forward from it, is the stepping out of the building, there's massive sort of benefits for you. I13 (Male; ≤39; SOC2)

While some individuals missed social interactions at work, others felt that less contact with their colleagues and/or the work environment reduced their stress levels. In addition, some reflected that home working made their underlying health conditions less visible and hence, less stigmatising.

There are fewer people around= less stressed. I can park my car when I use it for work. I saw less of my irritating colleagues. Q467 (Non-binary; 40-55; SOC2)

I was suffering from mental health issues, and this made me needing to work from home and take time off..[I now] stand out less from my colleagues. Q39 (Male; 40-55; SOC2)

Discussion

In this study of people living and working with long-term MSK conditions, we have explored the effects of the lockdown caused by COVID-19 on changes in work status and circumstances, and the perceived impact of these changes on health and wellbeing. People who continued working often reported significant anxiety about becoming infected at work and perceived unfairness compared to those who were furloughed, or could work at home. Many reported anxiety about finances and future chances of employment. In many cases, people found themselves working from home for the first time. Home working was not a panacea for all but offered some advantages and disadvantages in terms of impact on mental and physical health and physical activity. Most importantly, our findings point to some solutions to address the disability employment gap as we move beyond the pandemic to enable those working with MSK conditions to remain in work: suitable equipment; hybrid

For peer review only

home working; flexibility; relationship with and support from managers; and minimising the need to travel in traditional commuter times.

There are strengths and weaknesses to consider when interpreting these findings. 61% of questionnaire respondents and 16 interviewees worked in professional/associate professional roles, and fewer of our participants worked in lower paid jobs. We collected data over a six-month period which reflected varying degrees of COVID-19 public health measures both over that time period and based on where people lived. We did not capture longitudinal data, including the longer-term impact of changes to working practices. However, the study included people with both inflammatory and non-inflammatory MSK conditions in well-characterised cohorts of 'real world' patients (19) pre-defined by symptoms or diagnosis as opposed to convenience samples. Inflammatory arthritis and chronic pain are good exemplars of disability for work caused by the range of long-term conditions and therefore, these findings may be applicable to a wider range of people trying to work with chronic health conditions.

The COVID-19 pandemic has brought into sharp focus population inequalities in terms of the health, work, and finances of people (20). We found that, in those with MSK conditions, all these impacts coalesced, causing a complex relationship between socio-economic status, vulnerability to COVID-19 and risk of work-related exposure. As in other studies, individuals aged >56 years made up the highest proportion of people who had changed job/were furloughed/made redundant during COVID-19 (21). This may have important implications for older people with MSK conditions wishing to return to work, as older workers in particular report more difficulties gaining employment after losing a job (21). Other general population studies have found that those working in more manual/lower paid work were less likely to be able to work from home (22). However, for individuals with MSK conditions on immunosuppressant medication who were advised to shield and work from home, we have shown those working in manual, lower paid jobs (often public-facing roles with a higher risk of work-based exposure) were less likely to be able do so. The financial impacts of this were significant if individuals could not be furloughed. People reported having no choice but to continue working and described the anxiety caused in consequence. Recent evidence indicates increased work-related exposure to COVID-19 in those unable to work

For peer review only - http://bmjopen.bmj.com/site/about/guidelines.xhtml

from home and who are in closer proximity to other people or in direct contact with the public; often low-income jobs in service sectors, such as health or social care, transportation, cleaning and hospitality (23). A recent online survey of 2,003 disabled workers or workers who have a health condition or impairment and who were in work at the start of the pandemic in February 2021 by the Trades Union Congress (24), suggests that disability employment gap has widened as a result of COVID-19.

Many of the advantages and disadvantages of working from home highlighted by people with chronic MSK conditions in this study are similar to those reported by workers without health conditions during the pandemic (25). People learned to adapt and many were able to work effectively from home. People with MSK conditions valued flexibility to organise tasks and the freedom to make decisions about when they did their work from home. Similarly, 9 out of 10 workers in one national survey reported that they got at least as much, if not more, work done at home as in the office, with almost three quarters of employees saying that they wanted to adopt hybrid working arrangements in the future (25). For people with MSK conditions, we have already shown that commuting to work, and driving, can be a significant challenge for people with inflammatory arthritis (7, 26), and these findings suggest that, for many, reduced need for commuting was beneficial.

However, home working was not a panacea. Amongst those for whom exercise formed part of their daily commute, working from home worsened pain and stiffness. Moreover, some missed the sociability and benefits of collaboration offered by working in shared workspaces (25). Whilst many reported improvements in work-life balance, and more time for exercise, others struggled to balance working from home with domestic responsibilities and managing their MSK condition on top of this created additional burden. Interestingly, general population studies of workers have suggested people developed more musculoskeletal pain, and higher levels of fatigue and poor sleep early after lockdown, although things improved somewhat after a period of adjustment (25, 27). Our study also highlighted the importance of the relationship between home-working employees and their managers. In a recent study of UK workers, participants who had more frequent contact with their line manager and had a work station risk assessment whilst home-working reported better mental health and less musculoskeletal pain (25). Line managers play a key

For peer review only

role in supporting, motivating and engaging a remote or hybrid workforce, and employee wellbeing and line manager support is closely linked to productivity at work (25).

We already know that if people are out of the workplace for a long period that they are less likely to come back (28, 29). Loss of both routine and contact with the workplace has a detrimental impact on inactivity, isolation, well-being, as well as impaired self-image and career opportunities, physical fitness and confidence (30). Similarly, prolonged unemployment, for any reason, causes additional health problems. Those who lose their job suffer from worse mental health (31), poorer life expectancy (32), attend healthcare consultations more frequently with physical symptoms and report higher levels of pain (33). The significant numbers of people with long-term MSK conditions who were previously coping at work, and who have now found themselves out of the workplace for long periods because of the pandemic, may suffer similar consequences without additional support.

Conclusions

This study provides new insights into the impact of the COVID-19 pandemic on people living and working with MSK conditions within well-defined cohorts. It provides lessons to support those working with long-term MSK conditions, those with other long-term conditions and the wider working population to work well and remain in work. Flexible working arrangements such as home-working can not only make the workplace more inclusive for people with disabilities and health conditions but can also have wider benefits on their MSK health and wellbeing. These findings should encourage structural and organisational changes at the workplace to support people with long-term MSK conditions to work. There have however been concerns that individualised or complicated working patterns might not be sustainable within an organisation or might be perceived as unfair by co-workers (25, 34). However, flexibility and trust between employers and employees are the foundations of “good work” (35) and employers creating an open, flexible workplace experience better productivity and reduced staff turnover. The lockdown has provided a learning opportunity for both employees and employers to think creatively and shape new models of work that can accommodate everyone. The current study provides evidence of the value of some of these approaches when they are made available.

For peer review only

The pandemic has exposed pre-existing inequalities in socio-economic circumstances, health and work. Unfortunately, we have seen that people with long-term MSK conditions for whom flexibility was not possible, tended to be those from more socio-economically deprived backgrounds who have consequently been exposed to financial hardship or job loss or felt forced to ignore advice to shield and continue working, putting themselves at increased risk. Rheumatology services may see the consequences of this on work, health and finances for many years to come. However, some of the findings reported here may enable the rheumatology multi-disciplinary team to better support work participation for all patients with long-term MSK conditions and address the disability employment gap.

Data sharing statement

The data within the article which relate to the collection of BSR register data are owned by the BSR – access to these data are subject to application being made to the BSR: Registers (rheumatology.org.uk).

Contributorship statement

We are grateful to our patient partner Inga Wood for help with designing the interview schedule and for commenting on the manuscript and Lynne Laidlaw for help with designing questionnaire. The authors do not report any conflicts of interest. GJM conceived the idea for the study and all authors were involved in the detailed planning. LM, KS, and RH conducted the qualitative analysis with input from KWB. MB and GJ undertook the questionnaire analysis. LM and RH integrated questionnaire and quantitative findings, and KWB, KS and GJM contributed to interpretation of findings. RH and LM drafted the manuscript and all authors contributed important intellectual content via written comments.

For peer review only - http://bmjopen.bmj.com/site/about/guidelines.xhtml

References

1. Waddell GaB, A Kim. Is Work Good For Your Health and Well-Being? In: Pensions DfWa, editor.: The Stationary Office; 2006.
2. Black C. Working for a healthier tomorrow. Dame Carol Black's Review of the Health of Britain's Working Age Population. 2008.
3. Versus Arthritis. Working with Arthritis: Arthritis Research UK; 2016 [Available from: https://www.versusarthritis.org/media/2071/working-with-arthritis-policy-report.pdf.
4. Bridges S. Health Survey for England: Chapter 9 Chronic Pain. 2011. Contract No.: Report.
5. Price C, Hoggart B, Olukoga O, Williams A, Bottle A. National pain audit final report 2010–2012. London: Healthcare Quality Improvement Partnership, British Pain Society and Dr Foster Intelligence. 2012.
6. Macfarlane GJ, Shim J, Jones GT, Walker-Bone K, Pathan E, Dean LE. Identifying Persons with Axial Spondyloarthritis At Risk of Poor Work Outcome: Results from the British Society for Rheumatology Biologics Register. The Journal of rheumatology. 2019;46(2):145-52.
7. Hollick RJ, Stelfox K, Dean LE, Shim J, Walker-Bone K, Macfarlane GJ. Outcomes and treatment responses, including work productivity, among people with axial spondyloarthritis living in urban and rural areas: a mixed-methods study within a national register. Ann Rheum Dis. 2020;79(8):1055-62.
8. Macfarlane G, Hollick R, Morton L, Heddle M, Bachmair E, Anderson R, et al. The effect of COVID19 public health restrictions on the health of people with musculoskeletal conditions and symptoms: the CONTAIN study. . Rheumatology.
9. Rudwaleit M, van der Heijde D, Landewe R, Listing J, Akkoc N, Brandt J, et al. The development of Assessment of SpondyloArthritis international Society classification criteria for axial spondyloarthritis (part II): validation and final selection. Annals of the Rheumatic Diseases. 2009;68(6):777-83.
10. Taylor W, Gladman D, Helliwell P, Marchesoni A, Mease P, Mielants H. Classification criteria for psoriatic arthritis: development of new criteria from a large international study. Arthritis & Rheumatism: Official Journal of the American College of Rheumatology. 2006;54(8):2665-73.
11. Macfarlane GJ, Beasley M, Scott N, Chong H, McNamee P, McBeth J, et al. Maintaining musculoskeletal health using a behavioural therapy approach: a population-based randomised controlled trial (the MAmMOTH Study). Annals of the Rheumatic Diseases. 2021:annrheumdis-2020-219091.
12. Scottish index of multiple deprivation (SIMD) 2016.
13. English indices of deprivation (EID) 2015.
14. StatsWales. The Welsh Index of Multiple Deprivation 2014. .
15. Office for National S. SOC2010 volume 2: the structure and coding index.
16. Kelly A, Tymms K, Fallon K, Sumpton D, Tugwell P, Tunnicliffe D, et al. Qualitative Research in Rheumatology: An Overview of Methods and Contributions to Practice and Policy. J Rheumatol. 2021;48(1):6-15.
17. Braun V, Clarke V. Using thematic analysis in psychology. Qualitative Research in Psychology. 2006;3(2):77-101.
18. Braun V, Clarke V. To saturate or not to saturate? Questioning data saturation as a useful concept for thematic analysis and sample-size rationales. Qualitative Research in Sport, Exercise and Health. 2019;13(2):201-16.
19. Jones GT, Dean LE, Pathan E, Hollick RJ, Macfarlane GJ. Real-world evidence of TNF inhibition in axial spondyloarthritis: can we generalise the results from clinical trials? Annals of the Rheumatic Diseases. 2020;79:914-9.
20. Johnson P, Joyce R, Platt L. The IFS Deaton Review of Inequalities: a New Year's message. 2021. Contract No.: Report.
21. Bui TTM, Button P, Picciotti EG. Early Evidence on the Impact of Coronavirus Disease 2019 (COVID-19) and the Recession on Older Workers. Public Policy & Aging Report. 2020;30(4):154-9.

For peer review only - http://bmjopen.bmj.com/site/about/guidelines.xhtml

22. Adams-Prassl A, Boneva T, Golin M, Rauh C. Inequality in the impact of the coronovirus shock: new survey evidence for the UK. 2020. Contract No.: Report.
23. Industrial Injuries Advisory C. COVID-19 and occupation: IIAC position paper 48. 2021. Contract No.: Report.
24. Trades Union Congress (TUC). Disabled workers' experiences during the pandemic. A TUC Report 2021 [Available from: https://www.tuc.org.uk/sites/default/files/2021-06/Outline%20Report%20-%20Covid-19%20and%20Disabled%20Workers.pdf.
25. Parry J, Young Z, Bevan S, Veliziotis M, Baruch Y, Beigi M, et al. Working from Home under COVID-19 lockdown: Transitions and tensions, Work after Lockdown. 2021. Contract No.: Report.
26. Morton L, Macfarlane GJ, Jones G, Walker-Bone K, Hollick R. Driving difficulties in patients with axial spondyloarthritis: Results from the Scotland Registry for Ankylosing Spondylitis. Arthritis Care & Research. 2021.
27. Pieh C, Budimir S, Probst T. The effect of age, gender, income, work, and physical activity on mental health during coronavirus disease (COVID-19) lockdown in Austria. Journal of psychosomatic research. 2020;136:110186.
28. Black CaFD. Health at work - an independent review of sickness absence: The Stationery Office Limited; 2011 [Available from: https://assets.publishing.service.gov.uk/government/uploads/system/uploads/attachment_data/file/181060/health-at-work.pdf.
29. Waddell G, Burton AK. Occupational health guidelines for the management of low back pain at work: evidence review. Occupational Medicine. 2001;51(2):124-35.
30. Gustafsson K, Marklund S. Consequences of sickness presence and sickness absence on health and work ability: A Swedish prospective cohort study. International Journal of Occupational Medicine and Environmental Health. 2011;24(2):153-65.
31. Kposowa AJ. Unemployment and suicide: a cohort analysis of social factors predicting suicide in the US National Longitudinal Mortality Study. Psychological medicine. 2001;31(1):127.
32. Voss M, Nylen L, Floderus B, Diderichsen F, Terry PD. Unemployment and early cause-specific mortality: a study based on the Swedish twin registry. AmJPublic Health. 2004;94(12):2155-61.
33. Mason V. The prevalence of back pain in Great Britain.: Office of Population Censuses and surveys, HMSO; 1994.
34. Colella A. Coworker distributive fairness judgments of the workplace accommodation of employees with disabilities. Academy of management Review. 2001;26(1):100-16.
35. Taylor M, Marsh G, Nicol D, Broadbent P. Good work: the Taylor review of modern working practices. 2017. Contract No.: Report.

For peer review only

For peer review only - http://bmjopen.bmj.com/site/about/guidelines.xhtml

Standards for Reporting Qualitative Research (SRQR)*

http://www.equator-network.org/reporting-guidelines/srqr/

Title and abstract

	Page
**Title** - Concise description of the nature and topic of the study Identifying the study as qualitative or indicating the approach (e.g., ethnography, grounded theory) or data collection methods (e.g., interview, focus group) is recommended	1
**Abstract** - Summary of key elements of the study using the abstract format of the intended publication; typically includes background, purpose, methods, results, and conclusions	2

Introduction

**Problem formulation** - Description and significance of the problem/phenomenon studied; review of relevant theory and empirical work; problem statement	5-6
**Purpose or research question** - Purpose of the study and specific objectives or questions	6

Methods

**Qualitative approach and research paradigm** - Qualitative approach (e.g., ethnography, grounded theory, case study, phenomenology, narrative research) and guiding theory if appropriate; identifying the research paradigm (e.g., postpositivist, constructivist/ interpretivist) is also recommended; rationale**	9
**Researcher characteristics and reflexivity** - Researchers' characteristics that may influence the research, including personal attributes, qualifications/experience, relationship with participants, assumptions, and/or presuppositions; potential or actual interaction between researchers' characteristics and the research questions, approach, methods, results, and/or transferability	8
**Context** - Setting/site and salient contextual factors; rationale**	6-7
**Sampling strategy** - How and why research participants, documents, or events were selected; criteria for deciding when no further sampling was necessary (e.g., sampling saturation); rationale**	6-7; 9
**Ethical issues pertaining to human subjects** - Documentation of approval by an appropriate ethics review board and participant consent, or explanation for lack thereof; other confidentiality and data security issues	9
**Data collection methods** - Types of data collected; details of data collection procedures including (as appropriate) start and stop dates of data collection and analysis, iterative process, triangulation of sources/methods, and modification of procedures in response to evolving study findings; rationale**	7-8

For peer review only

**Data collection instruments and technologies** - Description of instruments (e.g., interview guides, questionnaires) and devices (e.g., audio recorders) used for data collection; if/how the instrument(s) changed over the course of the study	7-8
**Units of study** - Number and relevant characteristics of participants, documents, or events included in the study; level of participation (could be reported in results)	10-11
**Data processing** - Methods for processing data prior to and during analysis, including transcription, data entry, data management and security, verification of data integrity, data coding, and anonymization/de-identification of excerpts	8-9
**Data analysis** - Process by which inferences, themes, etc., were identified and developed, including the researchers involved in data analysis; usually references a specific paradigm or approach; rationale**	9
**Techniques to enhance trustworthiness** - Techniques to enhance trustworthiness and credibility of data analysis (e.g., member checking, audit trail, triangulation); rationale**	9

Results/findings

**Synthesis and interpretation** - Main findings (e.g., interpretations, inferences, and themes); might include development of a theory or model, or integration with prior research or theory	10-15
**Links to empirical data** - Evidence (e.g., quotes, field notes, text excerpts, photographs) to substantiate analytic findings	10-15

Discussion

**Integration with prior work, implications, transferability, and contribution(s) to the field -** Short summary of main findings; explanation of how findings and conclusions connect to, support, elaborate on, or challenge conclusions of earlier scholarship; discussion of scope of application/generalizability; identification of unique contribution(s) to scholarship in a discipline or field	16-19
**Limitations** - Trustworthiness and limitations of findings	16

Other

**Conflicts of interest** - Potential sources of influence or perceived influence on study conduct and conclusions; how these were managed	1
**Funding** - Sources of funding and other support; role of funders in data collection, interpretation, and reporting	1

*The authors created the SRQR by searching the literature to identify guidelines, reporting standards, and critical appraisal criteria for qualitative research; reviewing the reference lists of retrieved sources; and contacting experts to gain feedback. The SRQR aims to improve the transparency of all aspects of qualitative research by providing clear standards for reporting qualitative research.

For peer review only - http://bmjopen.bmj.com/site/about/guidelines.xhtml

**The rationale should briefly discuss the justification for choosing that theory, approach, method, or technique rather than other options available, the assumptions and limitations implicit in those choices, and how those choices influence study conclusions and transferability. As appropriate, the rationale for several items might be discussed together.

Reference:
O'Brien BC, Harris IB, Beckman TJ, Reed DA, Cook DA. **Standards for reporting qualitative research: a synthesis of recommendations.** *Academic Medicine*, Vol. 89, No. 9 / Sept 2014 DOI: 10.1097/ACM.0000000000000388

For peer review only

For peer review only - http://bmjopen.bmj.com/site/about/guidelines.xhtml
